# Comparative Analytical Performance and Clinical Agreement of Chemiluminescence Microparticle Immunoassay and Electrochemiluminescence Immunoassay for CA19-9: A Diagnostic Accuracy Study

**DOI:** 10.3390/diagnostics16162638

**Published:** 2026-08-19

**Authors:** Jongkonnee Wongpiyabovorn, Nasornthan Yutthakasaemsan, Sunida Vandelaer

**Affiliations:** Center of Excellence in Immune-Mediated Diseases, Department of Microbiology, Faculty of Medicine, Chulalongkorn University, Bangkok 10330, Thailand; kukkai.yut@gmail.com (N.Y.); sunida_80@hotmail.com (S.V.)

**Keywords:** reference value, cut-off value, ECLIA, CMIA, CA 19-9

## Abstract

**Background**: Carbohydrate antigen 19-9 (CA19-9) is widely used as a tumor marker for pancreatic and hepatobiliary malignancies. However, inter-assay variability may affect the interpretation of results. This study compared the analytical performance, reference values, diagnostic accuracy, and clinical concordance of CA19-9 measured by chemiluminescent microparticle immunoassay (CMIA) and electrochemiluminescence immunoassay (ECLIA) in a Thai population. **Methods**: Serum CA19-9 concentrations were measured using both CMIA and ECLIA in 398 subjects comprising 145 healthy individuals, 115 patients with benign conditions, and 138 patients with malignancies. Method comparison, reference interval assessment, receiver operating characteristic (ROC) analysis, and concordance analysis were performed. Population-specific cut-off values were established and compared with manufacturer-recommended thresholds. **Results**: CMIA produced modestly but significantly higher CA19-9 concentrations than ECLIA in healthy individuals, resulting in higher upper reference limits. The two assays showed strong correlations across the study population (R^2^ = 0.922). Among malignant conditions, CMIA generally yielded higher CA19-9 values than ECLIA, with significant differences observed in pancreatic cancer and cholangiocarcinoma (*p* < 0.001). Values in benign conditions were largely comparable, except in cirrhosis, where ECLIA produced higher values. Diagnostic performance was similar between CMIA and ECLIA (AUC 0.856 vs. 0.840). Population-derived cut-off values (34.945 kU/L for CMIA and 34.395 kU/L for ECLIA) showed diagnostic performance comparable to manufacturer-recommended cut-offs and improved inter-assay concordance, particularly in cholangiocarcinoma. **Conclusions**: CMIA and ECLIA demonstrated strong analytical correlation and comparable diagnostic performance for CA19-9. Nevertheless, systematic inter-assay differences indicate that results should be interpreted using assay-specific reference intervals and clinical decision thresholds. Population-specific cut-off values may further improve clinical interpretation, particularly in regions with a high prevalence of hepatobiliary malignancies.

## 1. Introduction

Carbohydrate antigen 19-9 (CA19-9) is a tumor-associated antigen identified by the monoclonal antibody 1116-NS-19-9 [1,2], which detects a carbohydrate epitope present on circulating glycoproteins. Previous research indicates that CA19-9 levels are frequently elevated in patients with a variety of gastrointestinal malignancies, including pancreatic, colorectal, gastric, and hepatic cancers [3,4,5,6,7,8,9,10,11,12,13,14,15,16]. However, its use for cancer screening is not supported by clinical evidence; instead, CA19-9 serves primarily as an adjunctive tool in the management of pancreatic cancer [2,17,18,19,20,21,22,23,24,25,26,27,28,29,30,31]. Elevated CA 19-9 levels have also been observed in metastatic disease and in benign conditions such as hepatitis, cirrhosis, pancreatitis, and other gastrointestinal disorders [2,32,33,34,35,36,37,38].

The initial clinical study on CA19-9, conducted using an immunoradiometric assay, established a cut-off value of 37 kU/L [39], which continues to be widely used in clinical practice. However, current automated immunoassays differ in analytical characteristics, including assay design, antibody specificity, and calibration, which may result in differences in measured CA19-9 concentrations [40,41,42]. In addition, the lack of an internationally recognized reference standard contributes to inter-assay variability, limiting the comparability of results obtained across different analytical platforms. Consequently, the universal application of a single cut-off value may lead to inconsistent interpretation of CA19-9 results, particularly when patients are monitored using different assay methods over time. Furthermore, CA19-9 concentrations may be influenced by population characteristics, including genetic background, disease spectrum, and the prevalence of benign conditions associated with elevated CA19-9 levels. For this reason, assay manufacturers recommend that individual laboratories verify or establish reference intervals using their local populations.

Nevertheless, evidence supporting assay-specific reference values and clinical decision limits remains limited in the Thai population, particularly for the two commonly used automated platforms, chemiluminescence microparticle immunoassay (CMIA) and electrochemiluminescence immunoassay (ECLIA). Therefore, this study aimed to establish population-specific reference values for CA19-9 using both CMIA and ECLIA and to evaluate their clinical validity in distinguishing benign from malignant gastrointestinal diseases.

## 2. Material and Methods

### 2.1. Patients and Samples

A total of 398 blood samples were included in the study, comprising 253 consecutive patients and 145 apparently healthy individuals. All consecutive eligible patients undergoing clinical evaluation or follow-up for gastrointestinal diseases at King Chulalongkorn Memorial Hospital during a three-month period were prospectively enrolled to minimize selection bias. Among the patient cohort, 115 patients were classified as having benign diseases after comprehensive clinical, laboratory, imaging, and/or pathological evaluation, despite having undergone CA19-9 testing for suspected malignancy. The remaining 138 patients had histologically or clinically confirmed malignancies, including pancreatic, cholangiocarcinoma, colorectal, ampulla of Vater cancer, and other malignancies. Patients were excluded if they had a serum creatinine > 2 mg/dL or serious concurrent conditions that could potentially influence serum CA19-9 concentration, including heart failure, renal failure, bleeding peptic ulcer, or intestinal paralysis.

Reference intervals were established using blood samples obtained from 145 apparently healthy adults (59 males and 86 females; aged ≥ 18 years) who attended routine health check-up. Individuals were excluded if they had diabetes mellitus requiring oral hypoglycemic agents or insulin therapy; chronic liver or kidney disease; hospitalization or critical illness within the preceding four weeks; blood donation within the previous three months; known HBV, HCV, or HIV infection; pregnancy or breastfeeding; or use of medications or supplements known to affect bone and calcium metabolism. Clinical information and laboratory data of all individuals were obtained through medical record review to verify eligibility. Liver biochemical parameters, including alkaline phosphatase (ALP), alanine aminotransferase (ALT), aspartate aminotransferase (AST), and total bilirubin, were reviewed to assess underlying hepatobiliary disorders.

The study was designed and conducted in accordance with the Clinical and Laboratory Standards Institute (CLSI) EP28-A3c guideline for reference interval establishment. The sample size for the healthy reference population (*n* = 145) satisfied the CLSI recommendation of at least 120 reference individuals for non-parametric estimation of reference intervals. The patient cohort represented all consecutive eligible cases presenting during the study period, reflecting routine clinical practice. The study protocol was approved by the Institutional Review Board, Faculty of Medicine, Chulalongkorn University (Approval No. 0032/2025; approved on 6 January 2025) and was conducted in accordance with the Declaration of Helsinki (1975, revised in 2013). Written informed consent was obtained from all participants before their enrollment in the study.

### 2.2. CA19-9 Testing

Blood samples were collected using Greiner Bio-One clotted (serum) blood collection tubes. The tubes were left at room temperature for 30 min and then centrifuged at 2730× *g* for 10 min. The upper serum was collected immediately and stored frozen at −20 °C for up to one month before CA19-9 testing. All serum samples, identified by barcode, were tested for CA19-9. Comparisons were made between 2 methods; CMIA on the Alinity i (Abbott Diagnostics, Chicago, IL, USA) and ECLIA on the COBAS e411 (Roche Diagnostics, Mannheim, Germany).

Both CMIA and ECLIA use monoclonal antibody 1116-NS-19-9 to detect CA19-9, but through different signal-generation mechanisms [43]. The CMIA assay for CA19-9 used microparticles coated with 1116-NS-19-9 mouse monoclonal antibody, and acridinium-labeled anti-mouse monoclonal 1116-NS-19-9 used as the conjugate. The chemiluminescent reaction of acridinium is triggered by sequential addition of a pre-trigger solution (hydrogen peroxide) and a trigger solution (sodium hydroxide). The ECLIA assay for CA19-9 used streptavidin-coated microparticles, biotin-coated 1116-NS-19-9 mouse monoclonal antibody, and ruthenium-labeled anti-mouse monoclonal 1116-NS-19-9 as the conjugate. The electrochemiluminescent reaction of ruthenium is triggered by the addition of tripropylamine (TPA) and transfer of the immune upon application of voltage; ruthenium and TPA react to produce light. The manufacturer-recommended cut-off values, as specified in the package insert are 37 kU/L for the Alinity i and 39 kU/L for COBAS e411. The analytical measuring intervals of CMIA and ECLIA are 0–1200 kU/L and 0–1000 kU/L, respectively. Samples with CA19-9 concentrations exceeding the analytical measuring range were automatically diluted 10-fold with the manufacturer’s universal diluent according to the manufacturer’s instructions and reanalyzed. Final reported concentrations were calculated after applying the appropriate dilution factor.

### 2.3. Statistical Analysis

Statistical analysis was performed using SPSS version 29.0 (IBM Corp., Armonk, NY, USA) and R software (version 4.5.2; *pROC* package). The upper reference limit for CA 19-9 was defined as the 97.5th percentile of values obtained from the healthy population. Log-transformed values were used for correlation and regression analyses due to the skewed distribution of CA19-9. Paired CA19-9 measurements obtained by CMIA and ECLIA were compared using the Wilcoxon signed-rank test. Pairwise method comparison between the two assays was performed using Passing–Bablok regression. Diagnostic performance was evaluated using receiver operating characteristic (ROC) curve analysis to compare sensitivity and specificity of the assays, and area under the ROC curves (AUCs) were compared using DeLong’s test. For subgroup analyses involving multiple pairwise comparisons, *p*-values were adjusted using the Bonferroni correction. A *p*-value < 0.05 was considered statistically significant.

## 3. Result

### 3.1. Reference Values

A total of 253 patient sera and 145 healthy individual sera were included in the analysis. Among the healthy individuals, 59 were males and 86 were females, with an age range of 18–62 years. In healthy subjects, CA19-9 concentrations measured by CMIA were modestly but significantly higher than those measured by ECLIA (mean ± SD: 11.55 ± 16.81 kU/L vs. 9.43 ± 9.45 kU/L, respectively; *p* = 0.028). Gender-specific analysis demonstrated significantly higher CA19-9 values in healthy females than in healthy males with both assays (CMIA: 14.35 ± 20.61 kU/L vs. 7.46 ± 7.09 kU/L; ECLIA: 10.59 ± 11.35 kU/L vs. 7.74 ± 5.31 kU/L; *p* = 0.024). In addition, the upper reference limits of CA19-9 determined by CMIA tended to be higher than those obtained by ECLIA. The 95th, 97.5th and 99th percentiles for CMIA versus ECLIA were 43.88 vs. 29.08 kU/ L, 59.81 vs. 37.99 kU/L and 110.83 vs. 56.69 kU/L, respectively (Table 1). In contrast, age-stratified analysis showed no significant differences in CA19-9 concentrations among healthy individuals.

### 3.2. Method Correlation and Concordance

**Figure 1:**
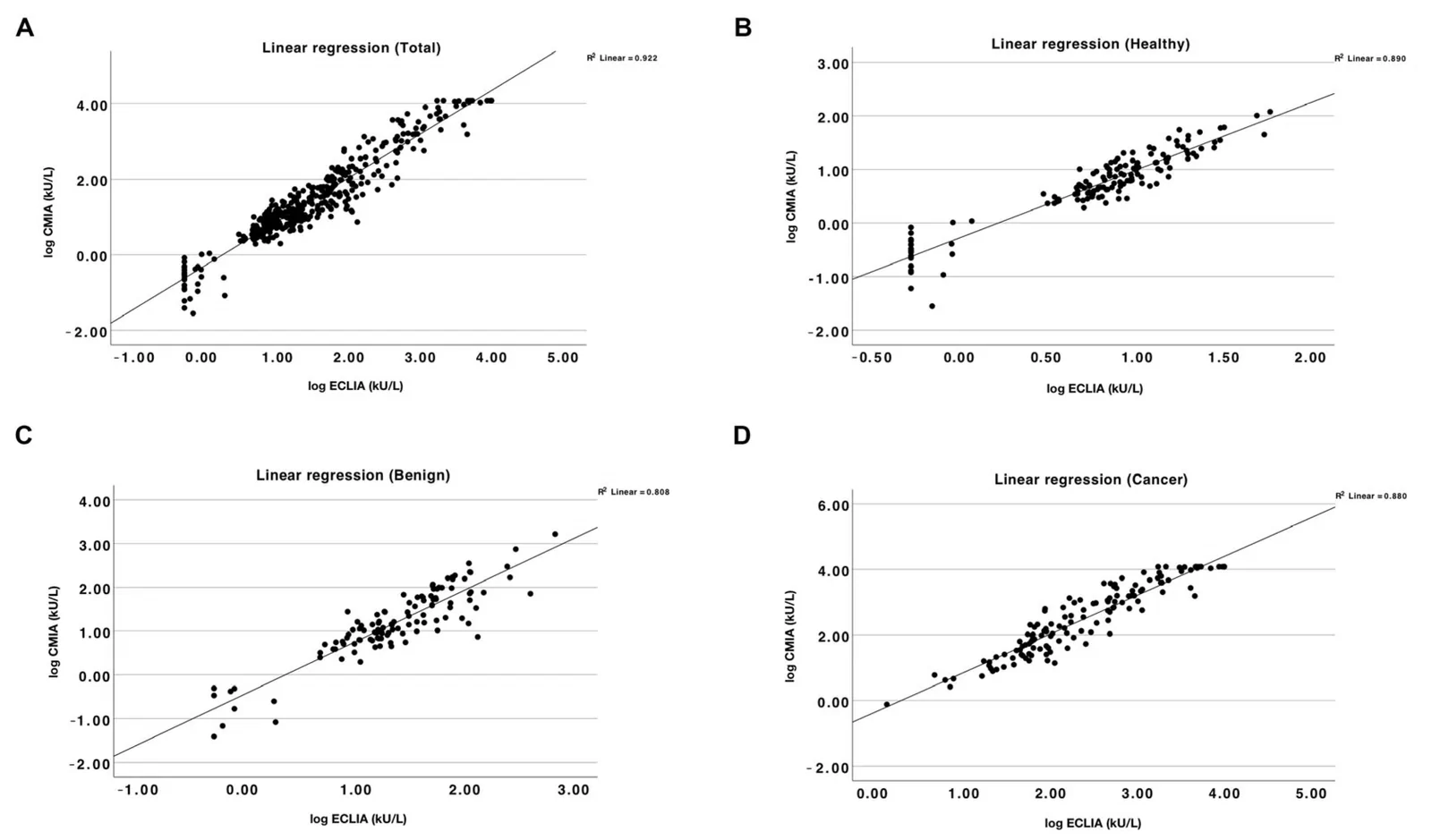
Correlation of CA19-9 concentrations measured by CMIA and ECLIA in the overall cohort ((**A**); *n* = 398, R^2^ = 0.922), healthy individuals ((**B**); *n* = 145, R^2^ = 0.890), patients with benign conditions ((**C**); *n* = 115, R^2^ = 0.808), and patients with malignant conditions ((**D**); *n* = 138, R^2^ = 0.880). A strong correlation was observed between the two assays across all study populations.

CA19-9 concentrations measured by CMIA and ECLIA showed strong correlation across all study populations. In the overall cohort (*n* = 398), a high degree of correlation was observed (R^2^ = 0.922). Subgroup analyses showed similarly strong correlations among healthy individuals (*n* = 145; R^2^ = 0.890), patients with benign conditions (*n* = 115; R^2^ = 0.808), and patients with malignancies (*n* = 138; R^2^ = 0.880) (Figure 1).

### 3.3. Clinical Validity

Serum CA19-9 concentrations from 138 patients with malignancies and 115 patients with various benign conditions were measured using both CMIA and ECLIA assays. The distribution of cancer types and benign conditions in the study population is summarized in Table 2 and Table 3. Across most malignancies, mean CA19-9 concentrations measured by CMIA tended to be higher than those obtained by ECLIA. Statistically significant differences were observed in pancreatic cancer and cholangiocarcinoma, with higher values detected by CMIA (pancreatic cancer: mean ± SD, 2794.89 ± 4001.05 kU/L vs. 1446.77 ± 2702.08 kU/L; *p* < 0.001; cholangiocarcinoma: 5371.55 ± 5302.12 kU/L vs. 2972.05 ± 3658.39 kU/L; *p* < 0.001) (Table 2). In contrast, no statistically significant differences in mean CA19-9 concentrations between the two assays were observed across benign conditions, with the exception of liver cirrhosis. In this subgroup, CA19-9 levels measured by ECLIA were significantly higher than those measured by CMIA (mean ± SD, 47.67 ± 59.36 kU/L vs. 28.02 ± 44.00 kU/L; *p* < 0.001) (Table 3).

**Figure 2:**
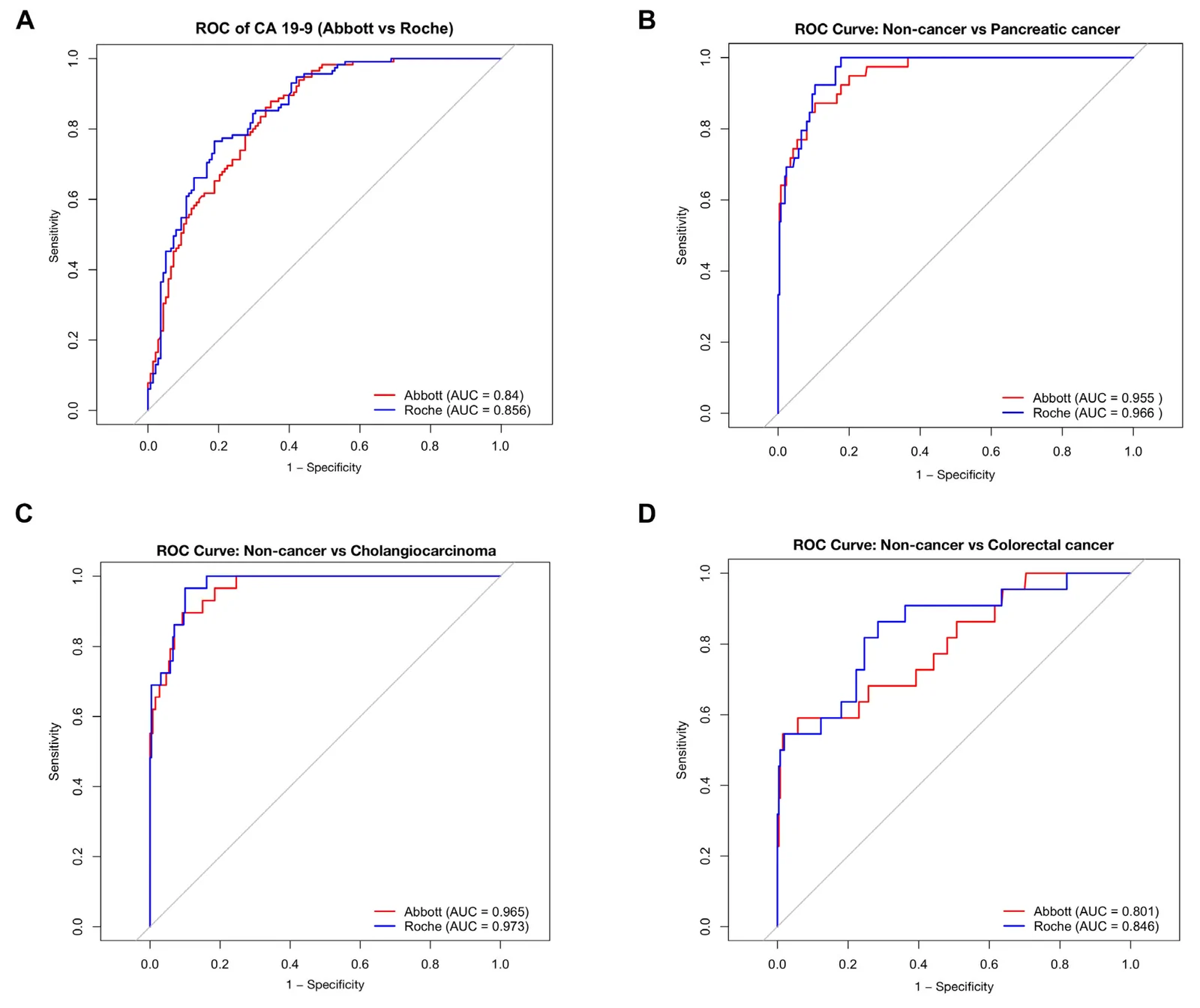
Receiver operating characteristic (ROC) curves of CA19-9 measured by CMIA and ECLIA for differentiating malignant from benign conditions in the overall study population (**A**), pancreatic cancer (**B**), cholangiocarcinoma (**C**), and colorectal cancer (**D**). The red and blue lines represent CMIA and ECLIA, respectively.

Receiver operating characteristic (ROC) curve analysis of the overall study population demonstrated comparable diagnostic performance for CA19-9 measured by CMIA and ECLIA in distinguishing malignant from benign conditions. The area under the curve (AUC) was 0.856 (95% CI, 0.811–0.901) for CMIA and 0.840 (95% CI, 0.792–0.887) for ECLIA, indicating similar overall diagnostic accuracy between the two assays (Figure 2A). Because the diagnostic performance of CA19-9 varies according to cancer type, disease-specific ROC analyses were subsequently performed. Both assays demonstrated excellent diagnostic performance for pancreatic cancer and cholangiocarcinoma. For pancreatic cancer, the AUCs were 0.955 (95% CI, 0.927–0.983) for CMIA and 0.964 (95% CI, 0.94–0.989) for ECLIA, whereas for cholangiocarcinoma the corresponding AUCs were 0.966 (95% CI, 0.946–0.986) and 0.973 (95% CI, 0.955–0.992), respectively. In contrast, diagnostic performance was more modest for colorectal cancer, with AUCs of 0.801 (95% CI, 0.695–0.908) for CMIA and 0.846 (95% CI, 0.753–0.939) for ECLIA (Figure 2B–D). Overall, both assays exhibited comparable diagnostic performance within each disease category, while the diagnostic accuracy of CA19-9 varied substantially across different malignancies.

Based on ROC analysis in this Thai population, the optimal cut-off value for CMIA was 34.945 kU/L, yielding a sensitivity of 79.7%, specificity of 93.1%, positive predictive value (PPV) of 91.67%, negative predictive value (NPV) of 82.82%, and overall accuracy of 86.57%. For ECLIA, an optimal cut-off value of 34.395 kU/L provided a sensitivity of 89.13%, specificity of 97.93%, PPV of 97.62%, NPV of 90.45%, and accuracy of 93.64%. The diagnostic performance achieved with these population-derived cut-off values was comparable to that obtained using the manufacturer-recommended thresholds of 37 kU/L for CMIA on the Alinity i system and 34 kU/L for ECLIA on the COBAS e411 platform (Table 4).

Using these population-derived cut-off values, concordance between CMIA and ECLIA was further evaluated and compared with that obtained using the manufacturer-recommended cut-offs (37 kU/L for CMIA and 34 kU/L for ECLIA). When manufacturer cut-offs were applied, assay concordance for benign conditions was 87.0% (κ = 0.723, *p* < 0.001), with higher concordance observed in malignant conditions (all cancers: 89.9%; pancreatic cancer: 92.3%; cholangiocarcinoma: 93.1%; κ = 0.629, *p* < 0.001). Application of the population-specific cut-off values resulted in improved overall concordance between the two assays. Concordance for benign conditions increased to 87.8% (κ = 0.742, *p* < 0.001), while concordance in malignant conditions was further enhanced, particularly in cholangiocarcinoma (all cancers: 90.6%; pancreatic cancer: 92.3%; cholangiocarcinoma: 96.6%; κ = 0.648, *p* < 0.001) (Table 5).

## 4. Discussion

To date, several available CA19-9 assays have been used to evaluate the utility of CA19-9 for screening, differential diagnosis, prognosis and monitoring of treatment reponse, particularly in patients with pancreatic cancer and cholangiocarcinoma [8,12,18,27,44,45,46,47,48,49,50,51]. Previous studies demonstrated variation in CA19-9 value among different assays [40,41,42]. However, there was no such study has been conducted in a Southeast Asian population, which has a high incidence of cholangiocarcinoma.

This study provides a comprehensive comparison of CA19-9 measurements obtained using CMIA and ECLIA platforms, focusing on reference values, analytical correlation, diagnostic performance, and assay concordance in a Thai population. Overall, our findings demonstrate strong analytical agreement and comparable clinical performance between the two assays, while highlighting clinically relevant differences in absolute CA19-9 concentrations and upper reference limits. These findings are consistent with several previous reports [39,42].

In healthy individuals, CA19-9 concentrations measured by CMIA were modestly but significantly higher than those obtained by ECLIA. Additionally, higher CA19-9 values observed in females compared with males across both platforms are consistent with previous reports and support consideration of sex-related biological variability when interpreting CA19-9 results [39,42,43,52]. Method comparison analysis demonstrated strong linear association between CMIA and ECLIA in the overall study population, as well as in clinically relevant subgroups, including healthy individuals, patients with benign conditions, and patients with malignancies. The consistently high coefficients of determination indicate good analytical correlation between the two assays. However, a strong correlation does not necessarily indicate that the assays are interchangeable, particularly given the systematic differences in absolute CA19-9 concentrations observed in this study.

Clinical validity analysis revealed that CA19-9 concentrations measured by CMIA tended to be higher than those measured by ECLIA across most malignancies. Statistically significant differences were observed in pancreatic cancer and cholangiocarcinoma, where CMIA yielded substantially higher values. These findings may reflect differences in assay calibration, antibody specificity, and the recognition of circulating CA19-9 isoforms. Furthermore, the lack of an internationally accepted reference standard for CA19-9 limits assay standardization. Consequently, differences in manufacturer-specific calibration systems and antibody design may result in systematic variation in measured concentrations. These findings highlight the importance of assay-specific interpretation. The use of assay-specific reference intervals together with externally validated clinical decision thresholds may improve the interpretation and clinical application of CA19-9 results. In contrast, CA19-9 concentrations in benign conditions were largely comparable between the two platforms, with the notable exception of liver cirrhosis, in which ECLIA produced significantly higher values. This observation is consistent with previous publications and may be explained by cholestasis-associated CA19-9 elevation and differential assay sensitivity to non-malignant antigen expression [1,32,38,39].

Despite these quantitative differences, ROC analysis demonstrated comparable diagnostic performance between CMIA and ECLIA for discriminating malignant from benign conditions. The AUC values for both assays were high and not meaningfully different, indicating similar overall diagnostic accuracy. Importantly, population-specific ROC analysis identified optimal cut-off values for both assays that were closely aligned with manufacturer-recommended thresholds and yielded strong sensitivity, specificity, and predictive values.

Application of population-derived cut-off values improved concordance between the two assays, particularly in malignant conditions. The highest concordance was observed in cholangiocarcinoma, a clinically relevant finding given the high prevalence of this malignancy in Thailand. These results suggest that locally derived cut-off values may enhance assay agreement and clinical interpretability, especially in regions with distinct disease epidemiology.

This study has several limitations that should be acknowledged. First, it was conducted at a single tertiary-care center in Thailand, which may limit the generalizability of the findings to other healthcare settings and populations. Second, although the overall healthy cohort (*n* = 145) met the minimum sample size recommended by CLSI EP28-A3c for establishing a nonparametric reference interval, the sex-specific subgroups did not reach the recommended minimum of 120 reference individuals per partition. Consequently, the estimated subgroup-specific 95th, 97.5th, and particularly the 99th percentile values should be interpreted with caution owing to increased sampling variability. These subgroup analyses were intended to provide preliminary descriptive data rather than definitive reference intervals and require validation in larger populations. Third, although the overall patient cohort was adequate for the primary analyses, several disease subgroups, particularly less common malignancies, included relatively few participants, limiting the statistical power for subgroup comparisons. Fourth, the ROC-derived cut-off values presented in this study represent internally derived estimates and should be interpreted as preliminary findings rather than validated clinical decision thresholds. Finally, because participants were recruited from routine clinical practice in a region with a high prevalence of cholangiocarcinoma, the findings may not be directly applicable to populations with different disease distributions. Therefore, multicenter studies involving larger and more diverse populations are warranted to validate these findings and establish broadly applicable assay-specific reference intervals and clinical decision thresholds.

An additional limitation is that several biological factors known to influence CA19-9 concentrations were not systematically evaluated. These include smoking status, benign biliary diseases, and inflammatory conditions, all of which may increase CA19-9 levels independently of malignancy. Furthermore, Lewis antigen status was not determined in this study. Because individuals with the Lewis-negative phenotype are unable to synthesize CA19-9 regardless of disease status, the absence of Lewis antigen typing may have influenced the interpretation of CA19-9 concentrations and the estimated diagnostic performance. Future studies incorporating these biological factors, particularly Lewis antigen status, would provide a more comprehensive assessment of assay performance and improve the clinical interpretation of CA19-9 measurements.

Our findings demonstrate that CMIA and ECLIA assays for CA19-9 showed good analytical correlation and comparable diagnostic performance. However, the observed systematic differences in measured values and upper reference limits indicate that results obtained from the two assays should be interpreted with assay-specific reference intervals and clinical decision thresholds rather than being used interchangeably. The use of assay- and population-specific reference intervals and cut-off values may help improve result interpretation, which is particularly relevant in clinical settings where CA19-9 is commonly used for the evaluation of hepatobiliary malignancies. Further studies evaluating clinically acceptable analytical variation and validating assay-specific decision thresholds are warranted to support their interchangeable use in clinical practice.

## Figures and Tables

**Table 1:** Gender specific distribution of CA19-9 concentrations (kU/L) determined with CMIA and ECLIA assays in 145 apparently healthy individuals.

Gender		Male	Female	Total
N (%)		59 (40.7%)	86 (59.30%)	145 (100.00%)
Assays/values				
Mean (kU/L)	CMIA	7.46 ± 7.09	14.35 ± 20.61	11.55 ± 16.81
	ECLIA	7.74 ± 5.31	10.59 ± 11.35	9.43 ± 9.45
Median (kU/L)	CMIA	4.59	6.26	5.81
	ECLIA	6.70	7.50	6.98
Range	CMIA	0.11–33.76	0.03–119.04	0.03–119.04
	ECLIA	0.50–22.05	0.50–58.70	0.50–58.70
95th percentile	CMIA	20.79	53.23	43.88
	ECLIA	18.52	30.35	29.08
97.5th percentile	CMIA	25.30	60.62	59.81
	ECLIA	19.72	46.65	37.99
99th percentile	CMIA	29.30	103.70	110.83
	ECLIA	20.68	54.93	56.69

**Table 2:** Distribution of CA 19-9 concentrations (kU/L) in 138 patients with malignancies determined with CMIA and ECLIA assays.

Cancer Type	*n* (%)	Mean ± SD (Range), kU/L of CA 19-9 Value		*p* Value
		CMIA	ECLIA	
Pancreas	39 (28.3)	2794.89 ± 4001.05 (13.77–12,001.00)	1446.77 ± 2702.08 (40.66–10,001.00)	<0.001
Cholangiocarcinoma	29 (21.0)	5371.55 ± 5302.12 (23.60–12,001.00)	2972.05 ± 3658.39 (44.66–10,001.00)	<0.001
Colorectal	22 (15.9)	2082.99 ± 3728.35 (4.61–12,001.00)	1189.14 ± 2352.03 (4.64–9563.00)	0.236
Ampulla	19 (13.8)	769.90 ± 2759.13 (0.76–12,001.00)	193.81 ± 445.57 (1.31–1736.00)	0.445
HCC	7 (5.1)	116.18 ± 129.76 (16.42–352.45)	149.09 ± 55.74 (92.37–255.40)	0.499
Gallbladder	6 (4.3)	3013.63 ± 4629.54 (53.66–12,001.00)	2206.92 ± 3879.23 (56.91–10,001.00)	0.345
Gastro-duodenal cancer	8(5.8)	1930.42 ± 4002.36 (16.23–11,698.97)	744.33 ± 1290.42 (56.87–3494)	0.36
Other diseases *	8 (5.8)	1086.87 ± 1273.29 (26.41–3331.84)	797.10 ± 1368.41 (56.74–4103.00)	0.327
Total	138 (100)			

* Lung (*n* = 4), Ovary (*n* = 3), Breast (*n* = 1).

**Table 3:** Distribution of CA 19-9 concentrations (kU/L) in 115 patients with various benign conditions determined with CMIA and ECLIA assays.

Benign Type	*n* (%)	Mean ± SD (Range), kU/L of CA 19-9 Value		*p* Value
		CMIA	ECLIA	
Cirrhosis	26 (22.6)	28.02 ± 44.00 (0.07–167.25)	47.67 ± 59.36 (0.60–261.90)	<0.001
Hepatic cyst	29 (25.2)	21.75 ± 25.48 (0.04–109.06)	19.69 ± 14.23 (0.50–51.75)	0.417
CBD obstruction	10 (8.7)	135.63 ± 234.24 (4.94–742.32)	90.79 ± 103.46 (5.22–296.00)	0.575
Fatty liver	6 (5.2)	14.73 ± 11.96 (4.48–34.67)	29.61 ± 22.70 (10.90–73.87)	0.116
Hepatitis	9 (7.8)	11.51 ± 11.84 (0.48–33.66)	31.81 ± 49.43 (0.77–127.90)	0.515
Pancreatic cyst	14 (12.2)	34.13 ± 37.84 (0.08–99.76)	33.35 ± 27.33 (0.71–76.20)	0.881
Pancreatitis	9 (7.8)	60.97 ± 56.41 (0.49–185.41)	79.52 ± 124.00 (0.50–403.50)	0.661
Benign gynecological mass	5 (4.3)	511.68 ± 641.23 (116.33–1648.98)	213.58 ± 262.67 (51.20–681.10)	0.43
Other diseases *	7 (6.1)	80.75 ± 55.43 (20.2–161.50)	67.32 ± 24.43 (42.24–114.4)	0.612
Total	115 (100)			

* Benign gallbladder mass (*n* = 1), breast mass (*n* = 1), Rheumatologic diseases (*n* = 3), respiratory diseases (*n* = 2).

**Table 4:** Diagnostic performance using population-derived cut-off values compared with manufacturer-recommended thresholds (37 kU/L for CMIA and 34 kU/L for ECLIA).

	Thai Population-Derived Cut-Off		Manufacturer-Recommended Cut-Off ^a^	
Assay	CMIA	ECLIA	CMIA	ECLIA
Cut-off (kU/L)^ a^	34.945	34.395	37	34
Sensitivity (95% CI)	0.7971 (0.72–0.86)	0.8913 (0.83–0.93)	0.7899 (0.71–0.85)	0.8913 (0.83–0.93)
Specificity (95% CI)	0.931 (0.88–0.96)	0.9793 (0.94–0.99)	0.9379 (0.89–0.97)	0.9793 (0.94–0.99)
PPV (95% CI)	0.9167 (0.85–0.95)	0.9762 (0.93–0.99)	0.9237 (0.86–0.96)	0.9762 (0.93–0.99)
NPV (95% CI)	0.8282 (0.76–0.88)	0.9045 (0.84–0.94)	0.8242 (0.76–0.87)	0.9045 (0.84–0.94)
Accuracy (95% CI)	0.8657 (0.82–0.90)	0.9364 (0.90–0.96)	0.8657 (0.82–0.90)	0.9364 (0.90–0.96)

PPV: positive predictive value. NPV: negative predictive value. ^a^ 97.5th percentile given by the manufacturer.

**Table 5:** Diagnostic concordance of CMIA and ECLIA using population-derived cut-off values compared with manufacturer-recommended thresholds for benign diseases, pancreatic cancer and cholangiocarcinoma.

	Cut-Off	Population	Concordance (%)	Kappa (*p* Value)
Manufacturer recommended cut-off	ECLIA 34 vs. CMIA 37	Benign	100/115 (87.00%)	0.723 (<0.001)
		All cancer	124/138 (89.90%)	0.629 (<0.001)
		Pancreas	36/39 (92.30%)	
		Cholangiocarcinoma	27/29 (93.10%)	
Thai population-derived cut-off	ECLIA 34.395 vs. CMIA 34.945	Benign	101/115 (87.80%)	0.742 (<0.001)
		All cancer	125/138 (90.60%)	0.648 (<0.001)
		Pancreas	36/39 (92.30%)	
		Cholangiocarcinoma	28/29 (96.60%)	

## Data Availability

The data are available from the first and corresponding author upon reasonable request.
